# Conchology Variations in Species Identification of Pachychilidae (Mollusca, Gastropoda, Cerithiodea) through Multivariate Analysis

**DOI:** 10.21315/tlsr2020.31.2.7

**Published:** 2020-08-06

**Authors:** Hadi Hamli, Norsyafiqah Abdul Hamed, Sharifah Hazirah Syed Azmai, Mohd Hanafi Idris

**Affiliations:** 1 Department of Animal Science and Fishery, Faculty of Agricultural Science and Forestry, Universiti Putra Malaysia Bintulu Sarawak Campus, Nyabau Road, P.O. Box 396, 97008 Bintulu, Sarawak, Malaysia; 2 Faculty of Fisheries and Food Sciences, Universiti Malaysia Terengganu, 21030 Kuala Terengganu, Terengganu Malaysia

**Keywords:** Freshwater, Gastropod, Morphometric, Multivariate Analysis, Pachychilidae, Air Tawar, Gastropod, Morfometrik, Analisis Multivariat, Pachychilidae

## Abstract

Pachychilidae is one of the freshwater gastropod family which was previously known under the Potamididae and Thiaridae families. Studies on freshwater gastropods especially on conchcology examinantions are still inadequate compared to marine gastropods. Morphological and morphometric studies of gastropods are practically used to identify and differentiate between species and necessary to complement molecular studies due to its low cost and tolerable resolving power of discrimination. The aim of the current study is to provide information on morphological and morphometric characteristics of Pachychilidae in Bintulu, Sarawak stream. A total of 20 individuals from each species of *Sulcospira testudinaria*, *Sulcospira schmidti, Brotia siamensis,* and *Tylomelania* sp*.* from Pachychilidae familiy were collected at three different sites from a small stream within the Bintulu area. Fourteen measurement of shell morphometrics were converted into proportioned ratios and analysed for univariate and multivariate analysis. Three shell morphometric (Aperture width, AW; Whorl width, WW2; and, Interior anterior length, AINL) of Pachychilidae indicated significant differences (*P* < 0.05) between species. However, multivariate analysis revealed that these shell morphometrics are pre-eminent factors to discriminate genus *Sulcospira, Brotia* and *Tylomelania*, as well as between *Sulcospira* species. This current study also suggests that these three characteristics are unique to *Sulcospira* species due to strong distinction among species. Findings on these three characteristics are significant for *Sulcospira* spp. as this study is the first shell morphometric report on the Pachychilidae species in Sarawak.

## Abstract (ms)

Pachychilidae adalah salah satu keluarga gastropod air tawar yang dahulu dikenali sebagai keluarga Potamididae dan Thiaridae. Kajian mengenai gastropod air tawar masih tidak mencukupi berbanding gastropod laut. Kajian morfologi dan morfometrik mengenai gastropod digunakan secara praktikal untuk mengenal pasti dan membezakan antara spesies dan diperlukan sebagai pelengkap untuk kajian molekul kerana kos yang rendah dan kebolehan pembezaan. Tujuan kajian ini adalah untuk menyediakan lebih banyak maklumat mengenai ciri-ciri morfologi dan morfometrik Pachychilidae di Sarawak. Sejumlah dua puluh sampel individu dari *Sulcospira testudinaria*, *S. schmidti*, *Brotia siamensis*, dan *Tylomelania* sp. daripada keluarga Pachychilidae dikumpulkan dari tiga kawasan sungai kecil yang berlainan dalam kawasan Bintulu. Empat belas ciri-ciri cengkerang yang diukur telah diubah menjadi nisbah yang dianggarkan dan dianalisis untuk analisa univariat dan multivariat. Tiga ciri cengkerang (AW, WW2, AINL) Pachychilidae menunjukkan perbezaan ketara (*P* < 0.05) antara spesies. Walau bagaimanapun, analisis multivariate mendedahkan bahawa ciri-ciri cengkerang ini merupakan faktor utama untuk membezakan spesies *Sulcospira* dari *Brotia* dan *Tylomelania*, serta antara spesies *Sulcospira*. Kajian ini menunjukkan bahawa ketiga-tiga ciri ini adalah unik kepada spesies *Sulcospir*a kerana perbezaan yang kuat antara spesies. Penemuan terhadap ciri-ciri tertentu ini penting bagi *Sulcospira* spp. kerana kajian ini adalah laporan pertama mengenai morfometrik cengkerang spesies Pachychilidae di Sarawak.

**Highlights**

- Four species of freshwater gastropod (*Sulcospira testudinaria*, *Sulcospira schmidti, Brotia siamensis, Tylomelania* sp*.*) under Pachychilidae family were analysed for shell differentiation using univariate and multivariate analysis.
- Three shell morphometric (Aperture width, Whorl width, Interior anterior length) of Pachychilidae indicated significant differences between *Sulcospira testudinaria, Sulcospira schmidti*, *Brotia siamensis* and *Tylomelania* sp.
- The study suggests that three shell characteristics are unique to *Sulcospira* species due to strong distinction among the species.

## INTRODUCTION

Freshwater gastropods can be classified into two groups which are Prosobranchia or gilled gastropods and Pulmonata or lunged gastropods. There are approximately more than 500 families of freshwater Prosobranchia and 150 families of freshwater Pulmonata found all over the world (Leveque *et al.* 2005). Family Pachychilidae is under the subclass Prosobranchia along with five other families within Sorbeconcha clade (Strong *et al*. 2008). Pachychilidae was previously known as Potamididae or Thiaridae (Poppe & Tagaro, 2006). Nevertheless, study on freshwater gastropods taxonomy is inadequate compared to marine gastropod.

Species discrimination can be done through shell morphology, reproduction characteristic (Köhler *et al*. 2004), molecular phylogenies (Köhler & Glaubrecht, 2003) and morphometric variation (Pramithasari *et al*. 2017). Shell morphology is commonly designed for evolutionary studies (Seilacher 1984; Palmer 1985). However, morphological studies on snails are still practically used to identify and differentiate between species. Morphological and morphometric studies of gastropod shells are important, even though present-day molecular technology is widely used to identify organisms precisely (Prioli *et al*. 2002). Variations between gastropod shells are not only caused by genetic changes, but also by environmental characteristics and existence of predators (Preston & Roberts 2007). In addition, genetic analysis is very costly compared to morphometric analysis that requires analysis of data using available software and hardware such as thermal cycler and DNA sequencer. The usage of morphometric analysis has obtained wide acceptance in the current biological scene as it is increasingly used as a necessary complement to molecular studies due to its reasonable cost and tolerable resolving power of discrimination (Doyle *et al.* 2018). Therefore, morphometric and morphology studies are faster compared to genetic analysis that usually takes more time to produce results. Species identification can be done *in-situ* based on morphological observation and this helps to avoid the misidentification of species.

Gastropods form significant roles in the ecology and promote worldwide economy. In ecology, gastropods are reliable water quality indicators and abundance of certain species may indicate or alarm of any water impoundment (Savic & Randjelovic 2016; Galan *et al.* 2015). Economically, many molluscs are edible thus became important due to human consumption and as the alternative source of protein (Hamli *et al.* 2012; Robinson 1999). Despite their ecological and economic importance, the world’s population of freshwater gastropods battles with loss and decline in number. Freshwater gastropods especially may encounter threats from habitat loss and degradation (Strong *et al.* 2008*).* In fact, a total number of 279 species had extinct whilst 1,794 threatened Gastropoda species were listed in the Red List of Threatened Species in 2012.

There are scarce number of studies about Pachychilidae in Malaysia, particularly on morphometric and morphological studies. While in Sarawak, there are handfuls of published studies about Pachychilidae and one of them was conducted by Hamli *et al*. (2013). Hamli *et al*. (2013) mentioned that there were 21 species of edible gastropods found in markets around Sarawak (Sibu, Mukah, Bintulu, Kuching, Miri, Limbang and Lawas) and two species are from Pachychilidae. Malaysia is one of the regions that is rich in biodiversity, however there is limited information can be found recorded on malacofauna communities especially freshwater gastropods, in Sarawak. Therefore, this study aims to determine on morphological and morphometric characteristics of Pachychilidae that essentially contribute to information in freshwater ecosystem.

## MATERIALS AND METHODS

### Study Area

**Figure 1:**
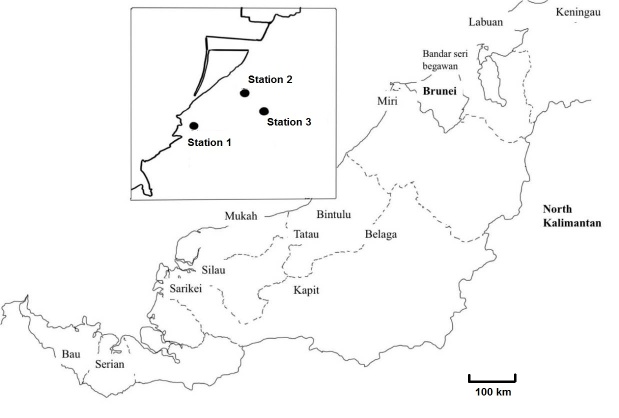
Study location of Pachychilidae in Bintulu, Sarawak.

A total of 20 individuals per species of *S. testudinaria* (von dem Busch 1842), *S. schmidti* (Martens 1908)*, B. siamensis* (Brot, 1886)*,* and *Tylomelania* sp. from the Pachychilidae family were collected at three different sites (N 03°12′55.3″ E 113°04′04.8″), (N 03°12.096′ E 113°02.978′), (N 03°12.469′ E 113°04.498′) from a small stream within the Bintulu area from October until December 2017 (Fig. 1).

## MORPHOLOGICAL STUDY

Samples were brought back to the laboratory for cleaning and visceral mass removal. Gastropod species was identified based on Köhler and Glaubrecht (2002), Köhler and Glaubrecht (2005), and Köhler and Simonis (2012). The gastropod shell was then photographed using camera (Cybershot DSCW810, Sony) from the apertural and abapertural view.

**Figure 2:**
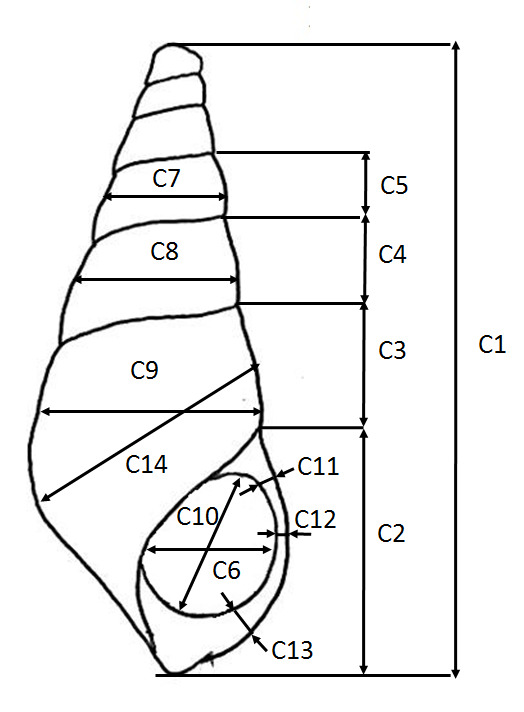
Shell characteristic used in the present study based on Sherely (1996). Detail shell morphometrics and characteristics as shown in Table 1.

A total of 15 shell morphometrics was measured using Mitutoyo digital callipers at 0.00 ± mm following Sherely’s (1996) (Fig. 2, Table 1). All shell morphometric were divided to shell length as proportion ratio to ensure no ontogeny bias.

## STATISTICAL ANALYSIS

The proportion ratio of all measured morphometrics were analysed using analysis of variance (ANOVA) with Tukey’s mean comparison test using statistical analysis computer software (SAS) version 9.4. Significant differences of all shell morphometrics between species were further tested with Principal Component Analysis (PCA) and Cluster analysis using Paleontological Statistics (PAST) Version 3.14.

## RESULTS

**Figure 3:**
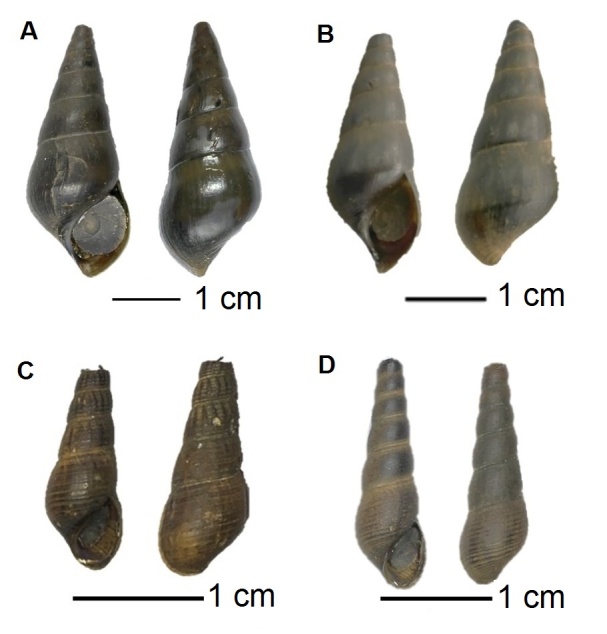
Pachychilidae species collected from Bintulu, Sarawak. (A) *S. testudinaria*; (B) *S. schmidti*; (C) *B. siamensis*; (D) *Tylomelania* sp.

Four species of Pachychilidae (Fig. 3) showed significant differences (*P* < 0.05) in eight shell morphometrics which were aperture length (AL), aperture width (AW), whorl width 1 (WW1), whorl width 2 (WW2), body whorl width (BWW), interior aperture length (AINL), lip thickness upper (LPU), and aperture whorl width (AWW) after being analysed with ANOVA (Table 2). However, only three shell characteristics, AW, WW, and AINL showed unique features for each of the species. *S. schmidti* displayed prominent aperture and whorl width compared to *S. testudinaria, B. siamensis,* and *Tylomelania* sp*.*

### Multivariate Analysis

**Figure 4:**
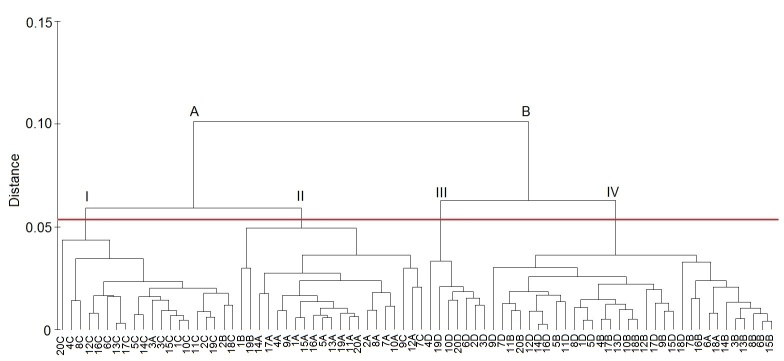
Cluster group of Pachychilidae based on three measurement of morphometric characters which are AW, WW2 and AINL. A = *S.testudinaria*, B = *B. siamensis*, C = *S. schmidti*, D = *Tylomelania* sp. N = Individual sample

Four and two clusters had been formed at 0.04 and 0.1 distances, respectively, based on the AW, WW2, and AINL characteristics (Fig. 4). Most samples of *S. schmidti* belong to Cluster I except one individual sample. However, one sample of *B. siamensis* and *S. testudinaria* were clustered along within Cluster I. Cluster II was representative of *S. testudinaria* due to 17 out of 20 samples belonging to this species. However, four samples within this cluster were associated to *B. siamensis* and *S. schmidti.* A total of 7 from 20 *Tylomelania* sp*.* samples were grouped under Cluster III and the rest of *Tylomelania* sp. clustered within Cluster IV together with *B. siamensis.* Therefore, Cluster IV was a combination of *Tylomelania* sp. and *B. siamensis.* Both *S. testudinaria* and *S. schmidti* were grouped in A which corresponds to the genus *Sulcospira.* However, *B. siamensis* and *Tylomelania* sp*.* were grouped under Cluster B due to overlapping shell morphometric.

**Figure 5:**
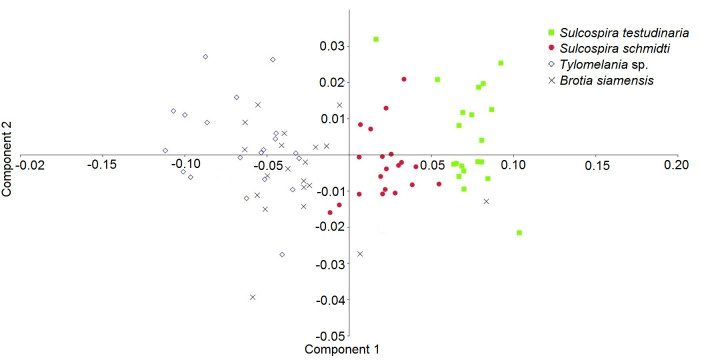
Scatter plot of PCA for Pachychilidae

The hierarchical clustering of Cluster A, B, I, II, III, and IV was similarly interpreted through scattered plot of PCA which indicated prominent separation between *S. testudinaria* and *S. schmidti* compared to *B. siamensis* and *Tylomelania* sp. (Fig. 5)*.* The principal components and shell morphometric showed a strong correlation for the first principal component with AW and AINL compared to WW2 (Table 3). The first principal component is primarily a measure of shell AINL (0.74215) which can be used as a tool to separate between *S. testudinaria*, *S. schmidti, B. siamensis*, and *Tylomelania* sp. Moreover, the second and third principal components mostly relate to shell AW and WW2, respectively for species distinction.

A proportion of AINL is considered a unique characteristic that can differentiate *S. schmidti* from the rest of Pachychilidae species found. Shell AW and WW2 can also be used to support the differentiation of species of Pachychilidae in the present study.

## DISCUSSION

Univariate analysis showed eight measurement of shell morphometric can be used to differentiate among species. However, only three measurement of shell morphometrics were proportionate to shell length (AL, WW2, AINL) and able to verify variation between four species of Pachychilidae. Köhler and Glaubrecht (2006) used five shell characteristics to discriminate between the two species of Pachychilidae, which are the minimum measurement of shell morphometric study. However, Köhler and Glaubrecht (2006) study was further supported by molecular work, thus make distinction among species more concrete. Most of the previous studies on gastropod shell morphometrics used at least four to thirteen shell characteristics (Dillon Jr. & Jacquemin 2015; Ismail & Elkarmi 2006; Köhler & Glaubrecht 2006; Chiu *et al.* 2002).

Common shell characteristics used in morphometric studies are shell length, shell width, body whorl length, penultimate whorl width, aperture length, and aperture width. Nevertheless, current findings only indicated that aperture width was able to differentiate between species compared to other common shell features used. Application of proportion ratio to this study is crucial to eliminate biases of sample age or ontogeny. Ontogeny is bias that affect morphology characteristic as organisms grow and develop (Herrel *et al.* 2016). The application has also been adapted for *Meretrix* spp. inner shell differentiation (Hamli *et al.* 2016). Therefore, it is important for shell measurements to be proportionate with shell length before further statistical analysis been done. Three shell characteristics AW, WW2 and AINL had indicated species distinction of *Sulcospira* genus compared to *Brotia* and *Tylomelania* genus. Furthermore, clear distinction between *S. schmidti* and *S. testudinari* indicates that AW, WW2, and AINL are strong differentiation characteristics for *Sulcospira* spp. However, differentiation between *Brotia* and *Tylomelania* was not apparently in this study. According to Köhler and Glaubrecht (2002), *Brotia* and *Tylomtelania* genus display similar characteristics which sometimes lead to both species being identified as *Brotia*. Additionally, both species have similar habitats and are distributed in the Southeast Asian region (Köhler *et al*. 2004).

*Brotia* and *Tylomelania* are able to be differentiated through reproductive biology characteristics, specifically a subhaemocoelic brood pouch and brood pouch formed by the pallial oviduct respectively (Köhler *et al.* 2004). Therefore, additional methods or other shell characteristics are needed to differentiate between *Brotia* and *Tylomelania.* Most morphometric analysis involves another method to increase accuracy of species discrimination. Generally, shell morphometric analysis is combined with molecular analysis using mitochondrial DNA (Potkamp *et al.* 2017; Köhler & Dames 2009). Other delineation studies are a combination of shell morphometrics with reproductive morphology (Köhler & Glaubrecht 2003), nervous system, radula, or alimentary system (Köhler & Glaubrecht 2006). Dillon and Jacquemin (2015) categorised shell morphometrics into two groups, traditional morphometrics and geometric morphometrics. The current study is categorised under traditional morphometrics which is based on linear measurement. Geometric morphometrics is a recent morphometric analysis method that is based on digitised landmarks of shells which was first introduced by Johnston *et al.* (1991). The use of geometric morphometrics has increased as an alternative for shell morphometric analysis that is widely accepted (Doyle *et al.* 2018; Dillon & Jacquemin 2015; Farman & Almukhtar 2015; Cruz *et al*. 2012).

## CONCLUSION

Based on the findings, application of more tools in morphometric studies must be emphasised to ensure strong differentiation between species and less dependence on just one practice. More discoveries on shell characteristics and additional techniques are needed to differentiate between *Brotia* sp. and *Tylomelania* sp. due to weak differentiation methods between these genera.

## Figures and Tables

**Table 1:** Abbreviations and descriptions of Pachychilidae shell morphometrics according to Sherely (2006)

Measurement number	Abbreviation	Description
C1	SL	Shell length: Maximum length of shell
C2	AL	Aperture length: maximum outside dimension of the aperture measured along an offset line to the right of the long axis of the shell
C3	WH1	Whorl height: measured from the intersection of the outside margin of the apertural lip and the edge of the periostracum on the first whorl that meets the apertural lip to the suture between adjacent whorls
C4	WH2	Whorl height: between top and bottom sutures of adjacent whorls
C5	WH3	Whorl height: between top and bottom sutures of adjacent whorls
C6	AW	Aperture width: maximum width of the aperture measured from the line demarcating the edge of the periostracum on the columella to the outer edge of the apertural lip
C7	WW1	Whorl width: dimensions were taken along lines parallel to the sutures from the midpoints of arcs formed by the outer edges of successive whorls
C8	WW2	Whorl width: dimensions were taken along lines parallel to the sutures from the midpoints of arcs formed by the outer edges of successive whorls
C9	BWW	Body Whorl width: dimensions were taken along lines parallel to the sutures from the midpoints of arcs formed by the outer edges of successive whorls
C10	AINL	Interior aperture length: maximum interior length of the aperture measured from the interior edges of the apertural lip
C11	LPL	Lip thickness: Lower raised areas of the apertural lip measured from the inside to the outer edges
C12	LPM	Lip thickness: Middle raised areas of the apertural lip measured from the inside to the outer edges
C13	LPU	Lip thickness: upper raised areas of the apertural lip measured from the inside to the outer edges
C14	AWW	Aperture whorl width: maximum width of the whorl above the aperture parallel with the columella

**Table 2:** Morphometric characters analysed by one way ANOVA with GLM.

Morphometric Characteristics	Proportion ratio for different species (Proportion ratio ± SE)					

	*S. testudinaria*	*S. schmidti*	*B. siamensis*	*Tylomelania* sp.	F value	*P*
AL	0.339 ±0.004^b^	0.399±0.005^a^	0.321±0.004^b^	0.321±0.006^b^	51.04	<0.0001
WH1	0.189±0.001^a^	0.190±0.002^a^	0.176±0.003^a^	0.175±0.003^a^	2.05	0.0935
WH2	0.136±0.001^a^	0.142±0.001^b^	0.139±0.002^ab^	0.138±0.003^ab^	4.22	0.0082
WH3	0.105±0.001^a^	0.108±0.001^a^	0.104±0.002^a^	0.111±0.002^a^	2.10	0.0869
AW	0.198±0.003^b^	0.225±0.002^a^	0.168±0.004 ^c^	0.154±0.002^d^	129.91	<0.0001
WW1	0.199±0.001^b^	0.215±0.002^a^	0.1870±0.003^c^	0.184±0.004^c^	28.20	<0.0001
WW2	0.255±0.002^b^	0.284±0.003^a^	0.234±0.00^3^c	0.222±0.00^4^d	70.31	<0.0001
BWW	0.325±0.002^b^	0.394±0.003^a^	0.296±0.005^c^	0.280±0.01^cd^	116.08	<0.0001
AINL	0.333±0.003^b^	0.3663±0.004^a^	0.299±0.006^c^	0.266±0.01 ^d^	72.12	<0.0001
LPL	0.007±0.000^a^	0.010±0.003^a^	0.008±0.000^a^	0.011±0.001^a^	1.59	0.1824
LPM	0.006±0.00^a^	0.008±0.002^a^	0.008±0.000^a^	0.009±0.001^a^	1.00	0.4120
LPU	0.006±0.00^b^	0.008±0.002^ab^	0.008±0.001^ab^	0.011±0.001^a^	3.65	0.0083
AWW	0.366±0.004^b^	0.436±0.004^a^	0.355±0.005^b^	0.368±0.005^b^	56.26	<0.0001

*Note*: Different superscripts indicate a significantly difference (*P* < 0.05) using Tukey mean comparison test.

**Table 3:** Variables loading on the first three component Pachychilidae species.

Variable	PC 1	PC 2	PC 3
AW	0.50233	0.36419	0.78424
WW2	0.4437	0.66989	−0.59529
AINL	0.74215	−0.647	−0.17492
Eigenvalue	0.003299	0.000161	0.000075
% variance	93.311	4.5613	2.1273

*Note*: Loading value near to 1 has a strong influence to component
